# Predictors of Outcomes after Correction of Acute Type A Aortic Dissection under Moderate Hypothermic Circulatory Arrest and Antegrade Cerebral Perfusion

**DOI:** 10.21470/1678-9741-2017-0123

**Published:** 2018

**Authors:** George Samanidis, Charalampos Katselis, Constantinos Contrafouris, Georgios Georgiopoulos, Ioannis Kriaras, Theofani Antoniou, Konstantinos Perreas

**Affiliations:** 1 First Department of Adult Cardiac Surgery, Onassis Cardiac Surgery Center, Athens, Greece.; 2 First Department of Cardiology, Hippokration Hospital, University of Athens, Athens, Greece.; 3 Department of Cardiac Surgery Intensive Care Unit, Onassis Cardiac Surgery Center, Athens, Greece.; 4 Department of Anesthesiology, Onassis Cardiac Surgery Center, Athens, Greece.

**Keywords:** Cerebrovascular Circulation, Hypothermia, Induced/Methods, Perfusion/Methods, Aneurysm, Dissecting/Surgery, Aortic Aneurysm/Surgery

## Abstract

**Introduction:**

Hypothermic circulatory arrest is widely used for correction of acute type A aortic dissection pathology. We present our experience of 45 consecutive patients operated in our unit with bilateral antegrade cerebral perfusion and moderate hypothermic circulatory arrest.

**Methods:**

Between January 2011 and April 2015, 45 consecutive patients were admitted for acute type A aortic dissection and operated emergently under moderate hypothermic circulatory arrest and bilateral antegrade cerebral perfusion.

**Results:**

Mean age was 58±11.4 years old. Median circulatory arrest time was 41.5 (30-54) minutes while the 30-day mortality and postoperative permanent neurological deficits rates were 6.7% and 13.3%, respectively. Unadjusted analysis revealed that the factors associated with 30-day mortality were: preoperative hemodynamic instability (OR: 14.8, 95% CI: 2.41, 90.6, *P*=0.004); and postoperative requirement for open sternum management (OR: 5.0, 95% CI: 1.041, 24.02, *P*=0.044) while preoperative hemodynamic instability (OR: 8.8, 95% CI: 1.41, 54.9, *P*=0.02) and postoperative sepsis or multiple organ dysfunction (OR: 13.6, 95% CI: 2.1, 89.9, *P*=0.007) were correlated with neurological dysfunction. By multivariable logistic regression analysis, postoperative sepsis and multiple organ dysfunction independently predicted (OR: 15.9, 95% CI: 1.05, 96.4, *P*=0.045) the incidence of severe postoperative neurological complication. During median follow-up of 6 (2-12) months, the survival rate was 86.7%.

**Conclusion:**

Bilateral antegrade cerebral perfusion and direct carotid perfusion for cardiopulmonary bypass, in the surgical treatment for correction of acute aortic dissection type A, is a valuable technique with low 30-day mortality rate. However, postoperative severe neurological dysfunctions remain an issue that warrants further research.

**Table:** 

Abbreviations, acronyms & symbols				
AAD	= Acute type A aortic dissection		HTK	= Histidine-tryptophan-ketoglutarate
ACP	= Antegrade cerebral perfusion		ICU	= Intensive care unit
AF	= Atrial fibrillation		MHCA/BACP	= Moderate hypothermic circulatory arrest and bilateral antegrade cerebral perfusion
BACP	= Bilateral antegrade cerebral perfusion		MHCA/UACP or BACP	= Moderate hypothermic circulatory arrest with unilateral or bilateral cerebral perfusion
CA	= Circulatory arrest		PND	= Permanent neurological dysfunctions
CCA	= Common carotid artery		RCP	= Retrograde cerebral perfusion
CNS	= Central nervous system		TIA	= Transient ischemic attack
CPB	= Cardiopulmonary bypass		TND	= Temporary neurological dysfunctions
CT	= Computed tomography		UACP	= Unilateral antegrade cerebral perfusion
DHCA	= Deep hypothermic circulatory arrest			
DHCA/UACP or BACP	= Deep hypothermic circulatory arrest with unilateral or bilateral cerebral perfusion			

## INTRODUCTION

Hypothermic circulatory arrest (CA) is widely used for correction of acute type A aortic dissection (AAD). In an attempt to establish a bloodless surgical field and ameliorate brain protection, which is more vulnerable in this case, a variety of techniques for cardiopulmonary bypass (CPB) and cerebral perfusion during CA have been suggested over the years^[1-3]^.

We present our experience of 45 consecutive patients who underwent AAD correction with moderate hypothermic CA and bilateral antegrade cerebral perfusion (MHCA/BACP) via the common carotid arteries.

## METHODS

### Study Population

Between January 2011 and April 2015, 45 consecutive patients underwent an emergency operation for type A AAD (with or without aortic root and valve involvement) with MHCA/BACP. The preoperative diagnosis was established with computed tomography (CT) of thoracic and abdominal aorta, including aortic arch branches. All preoperative, perioperative and postoperative data were recorded.

Two patients were operated with preoperative minor neurological dysfunction [transient ischemic attack (TIA)].

This study was carried out according to the principles outlined in the Declaration of Helsinki. It is a retrospective analysis approved by the hospital's institutional ethics committee (546/30-04-2015) and all patients gave their informed consent prior to the operation.

### Surgical Technique

All patients underwent an emergency surgical correction of AAD within 24 hours of admission. The briefly applied surgical technique consisted of standard median sternotomy and CPB via: 1) right or left carotid artery (connecting through a 10-mm synthetic graft)/bicaval cannulation in 42 patients and 2) femoral artery/bicaval cannulation in 3 patients. Gradual cooling of the body (1ºC/5 min) and alpha-stat (pH management) was utilized. Myocardial arrest and protection was achieved with retrograde delivery of histidine-tryptophan-ketoglutarate (HTK) crystalloid solution (Custodiol®).

Once CPB was instituted, the aortic cross-clamp was applied, the cooling initiated and, with the heart stopped, correction of the proximal ascending aorta ± aortic root was undertaken, including any other required correction.

When bladder temperature reached ≈ 23ºC, the head was packed in ice and the circulation, except for the carotids, was interrupted. CPB and antegrade cerebral perfusion (ACP) were achieved using synthetic graft (10 mm) in the right or left common carotid artery (CCA) and selective cannulation of the contralateral CCA. Where CBP was established with femoral artery/bicaval cannulation. ACP was performed with selective cannulation of both CCA. The ACP blood flow was 10 mL/kg/min (perfusion pressure around 50-70 mmHg, blood temperature ≈20ºC). The distal anastomosis in the proximal or middle aortic arch was performed. After heart de-airing and gradual rewarming, the patients were weaned from CPB.

### Degree of Postoperative Neurological Complications

Major postoperative neurological dysfunctions were evaluated by a neurologist and serial brain CT. In addition, neurological dysfunctions were divided in the four sub-groups: 1) no neurological dysfunction; 2) temporary neurological dysfunctions (TND): TIA, delirium and disorientation (<24 hours after extubation); 3) permanent neurological dysfunctions (PND): hemiplegia or paraplegia (>48 hours) that persisted after discharge at home; and 4) heavy neurological deficits (diffuse and irreversible brain damage or coma). Patients discharged were followed-up in regular intervals in outpatients' clinic with echocardiography and, if required, chest and brain CT scan.

### Statistical Analysis

Continuous variables are presented as means ± SD and categorical variables are expressed as percentages. Not normally distributed continuous variables are presented as medians (interquartile range). Univariate binary logistic regression analysis was performed to identify factors associated with postoperative mortality and/or neurological complications within 30 days from surgery. For 30-day mortality, the hospital length of stay and the presence of postoperative neurological complications were not evaluated as potential predictors to avoid selection bias (all subjects who died experienced postoperative neurological complications and presented less time of hospitalization). For postoperative neurological complications, where adequate number of events was yielded (n=9), significant univariate predictors were further incorporated into the final multivariable logistic regression model. To address the small sample size of our study, we implemented exact logistic regression and resampling techniques. Exact logistic regression produces more accurate inference in small samples because it does not depend on asymptotic results and conditional maximum probability estimates were sequentially calculated for each predictor in multivariable logistic models by temporarily conditioning out the other independent variables. Bootstrapping with 1000 replications was conducted to replicate bias-corrected confidence intervals of the significant determinants of the outcomes on interest in univariate and multivariable regression models. However, due to the limited number of observations, the reported effect sizes for certain variables are still characterized by wide confidence intervals.

Statistical analysis was conducted using STATA package, version 11.1 (StataCorp, College Station, Texas, USA). We deemed statistical significance at *P*<0.05.

## RESULTS

The cohort consisted of 34 male and 11 female patients. Mean age was 58±11.4 years. In 7 patients, haemodynamic instability and in 2 patients minor neurological deficits were noted preoperatively. Preoperative data and baseline demographic characteristics are shown in Table 1.

**Table 1:** Baseline demographic and preoperative data.

Preoperative data	No. of patients Total n=45
Sex	
Male	34 (75.6)
Female	11 (24.4)
Age (years)	58±11.4
Mean body surface area (m^2^)	2±0.24
Preoperative creatinine plasma (mg/dL), median (IQR)	0.9 (0.8-1.3)
Preoperative hemodynamic instability	7 (15.6)
Previous cardiac surgery operation	2 (4.4)
Preoperative NT-proBNP (pg/mL), median (IQR)	247 (114-816)
Preoperative D-dimer (µg/L), median (IQR)	6503 (3418-8610)

Data are presented as mean ± SD or median (IQR) for continuous variables and as number and percentage for categorical variables. IQR=interquartile range; NT-proBNP=N-terminal prohormone of brain natriuretic peptide; SD=standard deviation

CPB was instituted with: right or left carotid (connecting through a synthetic 10-mm graft)/bicaval cannulation in 42 patients and femoral artery/bicaval cannulation in 3 patients. Combined procedures were performed in 12 patients. Median circulatory arrest time was 41.5 (30-54) min and median minimum bladder temperature during circulatory arrest was 22.3 (20.8-24) ºC. Other perioperative details are presented in Table 2.

**Table 2:** Perioperative data.

Perioperative data	No. of patients Total n=45 (%)
Combined operations	12 (26.7)
Types of operations	
Interposition graft replacement	29 (64.4)
Interposition graft replacement + modified Bentall operation	5 (11.1)
Interposition graft replacement + aortic valve replacement	3 (6.7)
Interposition graft replacement + coronary bypass grafting	2 (4.4)
Interposition graft replacement + aortic arch replacement	6 (13.3)
Total circulatory arrest time (min), median (IQR)	41.5 (30-54)
Aortic cross-clamp time (min), median (IQR)	107 (92-130)
Cardiopulmonary bypass time (min), median (IQR)	211 (184-240)
Bladder temperature during circulatory arrest (ºC), median (IQR)	22.3 (20.8-24)
pH during circulatory arrest, median (IQR)	7.33 (7.29-7.35)

Data are presented as mean ± SD or median (IQR) for continuous variables and as number and percentage for categorical variables. IQR=interquartile range; SD=standard deviation

Major and minor postoperative complications and follow-up data such as postoperative atrial fibrillation (AF), postoperative open sternum, intensive care unit (ICU) stay, all postoperative neurological complications, 30-day mortality and other complications are listed in Table 3. During median follow-up of 6 (2-12) months the survival was 86.7% (39/45).

**Table 3:** Postoperative complications and follow-up data.

Postoperative complications and follow-up data	No. of Patients Total n=45 (%)
Atrial fibrillation	11 (24.4)
Permanent pacemaker	2 (4.4)
Pericardial effusion	5 (11.1)
Mechanical ventilation > 48 hours in intensive care unit	29 (64.4)
Postoperative open sternum	14 (31.1)
Postoperative acute kidney injury (increase postoperative >50 % of preoperative creatinine plasma)	23 (51.1)
Postoperative neurological dysfunction:	
No neurological dysfunction	36 (80)
Temporary neurological dysfunction	___
Permanent neurological dysfunction	6 (13.3)
Heavy neurological dysfunction	3 (6.7)
Postoperative transfusion of red blood cells (unit), median (range)	10 (2-38)
Postoperative transfusion of fresh frozen plasma (unit), median (range)	8 (3-36)
Postoperative creatinine plasma (mg/dL), median (IQR)	1.6 (1.3-2.3)
Postoperative stay in ICU (days), median (IQR)	8 (3-9)
Postoperative in-hospital stay (days), median (IQR)	12 (8-18)
Follow-up (months), median (IQR)	6 (2-12)
Overall mortality	6 (13.3)
30-days mortality	3 (6.7)
All cause deaths during median follow-up 6 (2-12) months	3 (6.7)

Data are presented as mean ± SD or median (IQR) for continuous variables and as number and percentage for categorical variables. Permanent neurological dysfunction (PND)=hemiplegia or paraplegia >48 hours after discharge at home. Heavy neurological deficits=diffuse and irreversible brain damage or coma. ICU=intensive care unit; IQR=interquartile range

Unadjusted analysis of factors associated with 30-day mortality showed: preoperative hemodynamic instability (OR: 14.8, 95% CI: 2.41, 90.6, *P*=0.004) and postoperative open sternum (OR: 5.0, 95% CI: 1.041, 24.0, *P*=0.044) (Table 4).

**Table 4:** Univariate risk factors for 30-day mortality.

Variable	No. of patients	30-day mortality	OR	95% CI	*P*-value
Overall	45	3 (6.7)			
Preoperative factors					
Age (years)	58±11.4		0.909	(0.805-1.01)	0.071*
Sex (female)	11 (26.19)	__	0.775	(0-7.7)	0.565
Preoperative hemodynamic instability	7 (15.6)	2 (4.4)	14.8	(2.41-90.6)	0.004*
Preoperative D-dimer			1.001	(0.999-1.001)	0.798
Preoperative NT-proBNP			1.002	(0.999-1.001)	0.658
Perioperative factors					
Arterial cannulation site (femoral artery)	3 (6.7)	__	3.74	(0-44.3)	0.999
Total aortic arch replacement	7 (15.6)	__	1.39	(0.14-15.9)	0.999
Combined operation	12 (26.7)	__	0.687	(0-6.8)	0.554
Cardiopulmonary bypass time			0.991	(0.955-1.019)	0.581
Aortic cross-clamp time			0.986	(0.933-1.028)	0.584
Total circulatory arrest time			0.957	(0.853-1.029)	0.374
Bladder temperature during circulatory arrest			1.29	(0.829-2.01)	0.227
Postoperative factors					
Postoperative open sternum	14 (31.1)	2 (4.4)	5	(1.041-24.0)	0.044*
Postoperative pericardial effusion	5 (11.1)	__	2.07	(0-22)	0.999
Postoperative atrial fibrillation	11 (24.4)	__	0.775	(0-7.7)	0.565
Postoperative stay in ICU			0.581	(0.299-1.127)	0.108

*P*-values are derived from exact univariate logistic regression.

* Confidence intervals are derived from bootstrapping with 1000 replications.

NT-proBNP=N-terminal prohormone of brain natriuretic peptide; ICU=intensive care unit

Furthermore, unadjusted analysis revealed that severe neurological dysfunction correlated with: preoperative hemodynamic instability (OR: 8.8, 95% CI: 1.41, 54.9, *P*=0.02) and postoperative sepsis or multiple organ dysfunction (OR: 13.6, 95% CI: 2.1, 89.9, *P*=0.007) while bladder temperature during circulatory arrest (˚C) (OR: 1.33, 95% CI: 0.980, 1.860, *P*=0.058) was marginally associated with this outcome (Table 5). Finally, by multivariate logistic regression analysis, postoperative sepsis/multiple organ dysfunction was associated (OR: 15.9, 95% CI: 1.05, 96.4, *P*=0.045) with the incidence of severe postoperative neurological complications (Table 6) independently of other significant univariate predictors (*i.e.* bladder temperature during circulatory arrest and presence of preoperative hemodynamic instability).

**Table 5:** Univariate risk factors for postoperative severe neurological complication.

Variable	No. of patients	Postoperative neurological complication	OR	95% CI	*P*-value
Overall	45 (100.0)				
Preoperative factors					
Age (years)	58±11.4		0.977	(0.915-1.042)	0.489
Sex (female)	11 (26.19)	1 (2.2)	0.338	(0.007-3.1)	0.416
Reoperation	2 (4.4)	__	1.64	(0-22)	0.999
Preoperative hemodynamic instability	7 (15.6)	4 (8.9)	8.8	(1.41-54.9)	0.02*
Preoperative NT-proBNP			0.999	(0.999-1.001)	0.621
Perioperative factors					
Arterial cannulation site (common carotid artery with graft)	42 (93.3)	8 (17.8)	0.480	(0.022-31)	0.999
Arterial cannulation site (femoral artery)	3 (6.7)	1 (2.2)	2.13	(0.426-10.6)	0.358
Total aortic arch replacement	7 (15.6)	2 (4.4)	1.75	(0.139-13.8)	0.614
Combined operation	12 (26.7)	2 (4.4)	0.748	(0.065-4.93)	0.999
Cardiopulmonary bypass time			1.002	(0.985-1.018)	0.805
Aortic cross-clamp time			1.01	(0.981-1.029)	0.673
Total circulatory arrest time			1.01	(0.974-1.037)	0.686
Bladder temperature during circulatory arrest			1.33	(0.980-1.86)	0.058*
Postoperative factors					
Postoperative open sternum	14 (31.1)	3 (6.7)	1.13	(0.155-6.6)	0.999
Postoperative acute kidney injury	23 (51.1)	6 (13.3)	2.2	(0.394-15.7)	0.459
Postoperative pericardial effusion	5 (11.1)	1 (2.2)	1	(0.018-12.2)	0.999
Postoperative permanent pacemaker implantation	2 (4.4)	__	1.64	(0-22)	0.999
Postoperative atrial fibrillation	11 (24.4)	3 (6.7)	1.73	(0.228-10.6)	0.666
Postoperative sepsis or multiple organ dysfunctions	6 (13.3)	4 (8.9)	13.6	(2.1-89.9)	0.007*
Postoperative stay in ICU			1.040	(0.966-1.12)	0.254
Postoperative hospital stay			1.031	(0.982-1.1)	0.180

*P*-values are derived from exact univariate logistic regression.

* Confidence intervals are derived from bootstrapping with 1000 replications.

NT-proBNP=N-terminal prohormone of brain natriuretic peptide; ICU=intensive care unit

**Table 6:** Multivariable logistic regression analysis for the main determinants of the incidence of postoperative severe neurological outcome (n=45).

Variable	OR	95% CI	*P*-value
Bladder temperature during circulatory arrest	1.28	0.885-1.89	0.183
Preoperative hemodynamic instability	5.5	0.454-77	0.221
Postoperative sepsis or multiple organ dysfunction	15.9	1.05-96.4	0.045*

*P*-values are derived from exact logistic regression.

## DISCUSSION

Publications of series on operations in the ascending aorta and hemiarch continue to discuss advantages and disadvantages amongst different techniques particularly in AAD pathology^[1-7]^. However, there is unanimity in that the most significant concern during this type of operations is the protection of the central nervous system (CNS) during the interval of hypothermic CA. The main issues that need to be addressed in these complex procedures are route of CPB establishment (site of arterial cannulation), core temperature management during operation and type of cerebral perfusion during CA with or without aortic arch repair.

### Arterial Cannulation Sites

The optimal arterial cannulation site during proximal aortic arch surgery has been widely discussed over the years. Direct cannulation of the ascending aorta in patients who have an aortic aneurysm is an accepted technique and supported by many authors^[8]^. On the other hand, in cases with acute and chronic aortic dissection, the approach of arterial cannulation is controversial. Many techniques have been suggested in these cases: ascending aortic cannulation, right axillary or subclavian artery, right or left carotid artery, right or left common femoral artery and even transapical aortic cannulation^[9-14]^. These studies have demonstrated a relatively similar 30-day mortality regardless of the cannulation technique, while the incidence of stroke varied widely between 3.8 and 21%. In 2010, Tiwari et al.^[15]^, after analysis of several studies, concluded that ascending aortic cannulation has promising results with a lower mortality rate, but a higher stroke rate in type A aortic dissections. In our study, the incidence of PND and 30-day mortality was 13.3% and 6.7%, respectively. Although the numbers of patients in our study are too small to draw safe conclusions, the survival rate seems to be very good, with good survival in the medium term. Neurological complications, however, according to the above remain an issue.

### Temperature Management during Hypothermic Circulatory Arrest

The goals of hypothermia during thoracic aortic surgery are reduction of brain metabolism and attenuation of CNS damage during CA. In 1975, Griepp et al.^[16]^ described four patients with successful outcome who underwent aortic arch replacement with prosthetic graft under deep hypothermic circulatory arrest (DHCA) with lowest esophageal temperature at 14ºC and lowest rectal temperature at 18ºC. Decreased mortality and neurological complications were achieved with cerebral perfusion during CA. Deep hypothermic circulatory arrest with retrograde cerebral perfusion (DHCA/RCP) (18ºC) allowed longer CA times and improved brain protection^[17]^. In 1991, Bachet et al.^[18]^ published the technique with MHCA/ACP during transverse aortic arch repair (core temperature 25-28ºC and brain perfused with blood cooled at 6-12ºC). This method created a favorable circumstance to perform more complex operations in the aortic arch, with simultaneous improvement of neurological and overall outcomes.

In 2011, the analysis of 1558 patients (GERAADA study) by Krüger et al.^[19]^ noted that relative increase in mortality was observed in patients with hypothermic CA (<15ºC) alone, even more so when CA arrest time exceeded 30 min. Estrera et al.^[20]^ used hypothermic CA (nasopharyngeal temperature 15-20ºC) with or without retrograde cerebral perfusion (RCP) and stroke rate and 30-day mortality were 2.3% and 10.4%, respectively. On the other hand, Urbanski et al.^[21]^ analyzed 347 patients who underwent non-emergent arch surgery under MHCA with rectal temperature (28-34ºC) utilizing ACP. Their 30-day mortality and overall postoperative neurological dysfunction were 0.9% and 3.2%, respectively. Perreas et al.^[22]^, in a retrospective analysis of 208 patients operated on with DHCA/RCP (temperature range 11-24ºC), concluded that the core temperature within the specific range was not a risk factor for 30-day mortality and severe neurological events. In another recent study, Zierer et al.^[4]^ noted that increase of the core temperature (28-30ºC) during CA with implementation ACP allowed more time (CA>90 min), more complex corrections of ascending aorta and aortic arch pathology with low incidence of postoperative complications (new postoperative neurological deficits were 7%). Leshnower et al.^[23]^ analyzed 500 patients who underwent a hemiarch replacement under mild (28.6ºC) *vs.* moderate (24.3ºC) hypothermic CA with unilateral ACP with operative mortality of 4.2% *vs.* 4.8% (*P*=0.80) and no differences in TND between the two groups. However, the incidence of PND was reduced in mild *vs.* moderate hypothermia (2.5% *vs.*7.2%) (*P*=0.01).

### Type of the cerebral protection and perfusion: DHCA alone, DHCA/RCP, deep hypothermic CA with unilateral or bilateral cerebral perfusion (DHCA/UACP or BACP), or moderate hypothermic CA with unilateral or bilateral cerebral perfusion (MHCA/UACP or BACP)?

In hemiarch with or without total aortic arch replacement, with or without stent grafting, the question remains: which method of brain protection is most effective and safe during these complex operations, particularly in patients with AAD?

Usui et al.^[1]^ analyzed 2792 patients and found no differences between the ACP and RCP groups in 30-day mortality (3.4% *vs.* 2.4%) and stroke rate (5% *vs.* 3%), but in the subgroup with RCP higher incidence of transient neurological dysfunction (5.8%) was observed. Misfeld et al.^[2]^ divided 636 patients who underwent aortic arch surgery in four groups: UACP, bilateral ACP (BACP), DHCA/RCP and DHCA only. The study showed that early mortality and five-year survival were not different between the surgical groups, but stroke rate was different in patients who did not receive ACP (*P*=0.035). Urbanski et al.^[21]^ presented results of non-emergent aortic arch surgery using mild to moderate hypothermic CA (31.5±1.6ºC) with ACP (blood temperature 28ºC) in 347 patients. The results show that 30-day mortality was 0.9%, and PND and TND observed in 0.9% and 2.3%, respectively. On the other hand, Estrera et al.^[20]^ reported the study with 1107 patients who operated under DHCA/RCP in 82% of cases, and the results were 30-day mortality and stroke rate occurred 10.4% and 2.8%, respectively. After comparative analysis of 1558 patients with AAD, Krüger et al.^[19]^ concluded that 30-day mortality was higher in the DHCA group (19.4%) than in the BACP (15.9%) and UACP (13.9%) groups (*P*<0.05). The same study noted that PND were: DHCA-14.9%, BACP-14.1% and UACP-12.6%. The most recent results from the Japanese AAD database (2016) repair with ACP *vs.* RCP did not show significant differences regarding mortality and postoperative neurological dysfunctions rates (11.2% *vs.* 9.7%)^[3]^. Nowadays, many studies discuss the incidence of stroke and mortality rate after AAD repair with hemiarch *versus* hemiarch plus total arch replacement (with or without antegrade stent grafting)^[3,24-26]^. After analysis, the results show that the rates of mortality and stroke are similar between the two groups. However, it is possibly relevant regarding false lumen thrombosis rate and reduction of late reoperation rate in aortic arch and descending aorta. In 2015, Di Bartolomeo et al.^[26]^, in a review of AAD type A repair with frozen elephant trunk technique, noted that mortality and postoperative stroke range were 0-27.7% and 0-12%, respectively. In addition, in the same study, the authors revealed that the spinal cord injury rate ranged from 0 to 13.8%. It is evident that postoperative neurological complications range in the literature from 0 to 39.7%.

## CONCLUSION

In aortic arch surgery for AAD, the total CA can be avoided by maintaining cerebral perfusion. Thus, continuous cerebral perfusion via UACP or BACP resulted in a reduction of postoperative mortality. However, the postoperative severe neurological complications remain at high frequency, requiring further refinement of this technique. Reduction of these major postoperative complications should be explored in multicenter studies.

### Limitations

This is clearly a retrospective analysis with a small cohort, albeit with consecutive patients from a single unit. Two patients with preoperative TIA were included in our study with possible impact on severe postoperative neurological complications. Furthermore, most patients with AAD were operated emergently without accurate preoperative neurological assessment and without preoperative brain CT. On the other hand, more extensive follow-up is required to demonstrate potential improvements in the long-term outcomes.

**Table:** 

Authors' roles & responsibilities	
GS	Took part in the care of the patients and contributed equally in data collection and manuscript preparation; final approval of the version to be published
CK	Took part in the care of the patients and contributed equally in data collection and manuscript preparation; final approval of the version to be published
CC	Took part in the care of the patients and contributed equally in data collection and manuscript preparation; final approval of the version to be published
GG	Contributed in the statistical analysis of the data; final approval of the version to be published
IK	Took part in the care of the patients and contributed equally in data collection and manuscript preparation; final approval of the version to be published
TA	Took part in the care of the patients and contributed equally in data collection and manuscript preparation; final approval of the version to be published
KP	Supervision of this report; final approval of the version to be published
